# Porcine Epidemic Diarrhea Virus: Etiology, Epidemiology, Antigenicity, and Control Strategies in China

**DOI:** 10.3390/ani14020294

**Published:** 2024-01-17

**Authors:** Jianlin Lei, Yongqiang Miao, Wenrui Bi, Chaohui Xiang, Wei Li, Riteng Zhang, Qian Li, Zengqi Yang

**Affiliations:** 1 College of Agriculture and Forestry Science and Technology, Longdong University, Qingyang 745000, China; liqian08@lzu.edu.cn; 2 College of Veterinary Medicine, Northwest A&F University, Yangling 712100, China; 2020065012@nwafu.edu.cn (Y.M.); wenruibi@nwafu.edu.cn (W.B.); chovy@nwafu.edu.cn (C.X.); 1546280879@nwafu.edu.cn (W.L.); 2016107065@njau.edu.cn (R.Z.); yzq8162@126.com (Z.Y.)

**Keywords:** porcine epidemic diarrhea virus, enteric coronavirus, highly pathogenic variants, etiology, epidemiology, antigenicity, control strategies

## Abstract

**Simple Summary:**

Since 2010, the highly pathogenic variants of PEDV have spread widely around the world, posing a huge threat to the pig industry. The lack of an efficient vaccine at present makes it challenging to prevent PEDV from spreading throughout China. In this review, we outline the etiology, epidemiology, and antigenicity of PEDV and propose control strategies. We hope to provide basic information for an understanding of PEDV etiology and the formulation of effective control measures.

**Abstract:**

Porcine epidemic diarrhea virus (PEDV) is a porcine enteric coronavirus, which is one of the main causative agents of porcine epidemic diarrhea (PED), with 100% morbidity and 80–100% mortality in neonatal piglets. Since 2010, large-scale PED caused by highly pathogenic variants of PEDV has occurred successively in China and other countries in the world, posing a great threat to the global pig industry. It has been demonstrated in many investigations that the classic attenuated vaccine strain, PEDV CV777, is insufficient to fully protect against the PEDV variants. Moreover, the maternally derived antibodies elicited by inactivated vaccines also cannot completely protect piglets from infection. In addition, feedback feeding poses a risk of periodic PEDV recurrence in pig farms, making it challenging to successfully limit the spread of PEDV in China. This review focuses on the etiology, epidemiology, antigenicity, and control strategies of PEDV in China and provides information for the formulation of effective control measures.

## 1. Introduction

Coronaviruses (CoVs) have single-stranded, positive-sense RNA viruses with the largest known genomes among all RNA viruses, ranging from 26 to 32 kb, and they are a family of viruses (*Alpha-*, *Beta-*, *Gamma-*, and *Deltacoronavirus*) that can cause major diseases in humans, such as severe acute respiratory syndrome (SARS), Middle East respiratory syndrome (MERS), and the recent outbreak of SARS-CoV-2 [1,2,3]. In pigs, several different CoVs have been identified, including four *Alphacoronaviruses*: porcine epidemic diarrhea virus (PEDV), transmissible gastroenteritis virus (TGEV), porcine respiratory coronavirus (PRCV), swine acute diarrhea syndrome-coronavirus (SADS-CoV), one *Betacoronavirus*: porcine hemagglutinating encephalomyelitis virus (PHEV), and a porcine *Deltacoronavirus* (PDCoV) [4]. Porcine CoVs have caused significant economic losses to the global pig industry due to their high mortality in neonatal piglets. It is noteworthy that there is the potential for zoonotic transmission because porcine CoVs continue to adapt and evolve in their hosts, changing their tropism. Recently, a study revealed that PDCoVs were identified in Haitian children with acute undifferentiated febrile illness, and the flexibility of the protein and its interaction with the host cell receptor may be impacted by modifications to the spike S1 subunit, which contains the receptor-binding domain [5]. Therefore, the zoonotic transmission of porcine CoVs could pose a threat to human health.

PEDV was originally discovered in the UK in 1971 and then spread to other European countries, where it occasionally broke out in the latter half of the 20th century [6,7]. In China, a comparable case of diarrhea caused by PEDV was initially documented in 1973, and PEDV was discovered in 1984 [8]. Since 2010, a pervasive outbreak of PED has afflicted the southern regions of China, impacting pigs across all age groups. Notably, the PEDV spread rapidly nationwide, and the mortality rate among neonatal piglets approached 100% [9,10]. Following the first reports of PEDV in the United States in 2013, a highly pathogenic PEDV emerged and quickly infected pig populations [11,12]. The virus then quickly spread throughout the pig industry in Europe, North America, and Asia, posing serious economic threats to the pig industry worldwide [13,14,15,16]. Recently, according to the statistics of major animal diseases in China from April to September 2023, the number of reported cases of porcine epidemic diarrhea (PED) has ranked first among class II animal diseases (http://www.moa.gov.cn/was5/web/search) (accessed on 25 October 2023). Although there are several pathogens that might cause PED, including other porcine CoVs, PEDV is the major causative agent of PED in China [17]. These data suggest that PEDV has spread widely throughout China and has developed into a significant disease that is obstructing the regular growth of the pig industry. In order to provide information that may help in infection control, this review focuses on the etiology, epidemic status, antigenicity, and preventative and control strategies of PEDV in China.

## 2. Etiology

### 2.1. PEDV Genome and Functions

PEDV is a single-stranded positive-sense RNA virus that belongs to the *alphacoronavirus* genus. It has a diameter of 95–190 nm, a characteristic nested crown, and a genome size of approximately 28 kb [18]. Seven overlapping open reading frames in the PEDV genome encode the replicase (ORF1a, 1b) nonstructural proteins and the accessory protein ORF3, the spike (S), the envelope (E), the membrane (M), and the nucleocapsid (N) structural proteins [18]. As the major envelope glycoprotein, the S protein promotes the entry of virions into the cell and gives the viral family name its corona-like appearance in electron micrographs. It comprises five domains: a signal peptide; an S1 region that aids in the attachment of virions to cellular receptors; an S2 region that mediates the fusion of the virus with host cells; a transmembrane domain; and a cytoplasmic tail. The S1 domain is further divided into two sub-domains: an N terminal domain and a C terminal domain, which have the potential to be receptor-binding domains [19]. The capacity of the S protein to bind to a receptor and its role in viral entry determine PEDV invasion and release, host range, tissue tropism, cross-species transmission, and trypsin-dependent proliferation [20]. Importantly, the S protein is the major target of induced neutralizing antibodies, and six neutralizing epitopes have been identified: the S1^0^ (aa 19–220) [21], S1^A^ (aa 435–485) [22], COE (aa 499–638) [23], SS2 (aa 748–755), SS6 (aa 764–771) [24], and 2C10 (aa 1368–1374) [25]. In addition, the PEDV S gene is very genetically diverse and is prone to mutation, and it is frequently utilized to evaluate the virulence and genetic variety of the strains that are circulating in the field [26,27]. The M protein, a component of the viral envelope, participates in the assembly and release of viral particles [28]. Furthermore, the recombinant PEDV M protein is used as the antigen in the indirect enzyme-linked immunosorbent assay (ELISA), which has great sensitivity and specificity in identifying PEDV antibodies [29]. Numerous activities in the viral life cycle have been linked to the N protein, such as regulating the production of viral RNA, encasing viral RNA in helical nucleocapsids, and assembling virions [29]. The N protein’s suppression of the host response might account for the increased PEDV replication in Vero E6 cells that overexpress the protein [30]. By delaying the S-phase cell cycle and inhibiting NF-κB from moving to the nucleus during the host cell confronting process, the PEDV N protein suppressed the formation of IFN-λ in IPEC-J2 cells [29,31]. The two polyproteins, pp1a and pp1ab, that are encoded by ORF1a and ORF1b are processed into 16 nonstructural proteins (nsps) that are crucial for the replication and synthesis of the viral genome [32]. As an accessory protein, the ORF3 involved in viral infection, which could promote the formation of vesicles, prolonged the S phase in target cells and accelerated the development of the attenuated strain that produced a truncated version of the ORF3 [33].

### 2.2. Emergence of PEDV Strains in China

When the first cases of PED were reported in the UK in 1971, the phrase “epidemic viral diarrhea” (EVD) was coined [34]. Many European countries were impacted by a similar viral diarrhea outbreak in 1976, known as EVD II [35]. One of the EVD samples those Belgian researchers collected in 1978 was identified experimentally as CV777, a new coronavirus strain that causes diarrhea in pigs [6,36]. The disease was collectively named “porcine epidemic diarrhea” (PED) in 1982 [37]. PED reports have been provided on a regular basis in China since 1973. The presence of PEDV in China was not confirmed until 1984 when the causative agent was discovered by utilizing fluorescently labeled antibodies and serum neutralization assays [9]. Between 1984 and the beginning of 2010, PEDV-related epidemics were mostly sporadic or there were local epidemics in some provinces. However, in 2010, a large-scale PED outbreak occurred in southern China [9,10]. Multiple studies have confirmed that emerging PEDV is the culprit of PED in China, and PEDV has mutated compared with previously prevalent strains [9,38,39].

**Figure 1:**
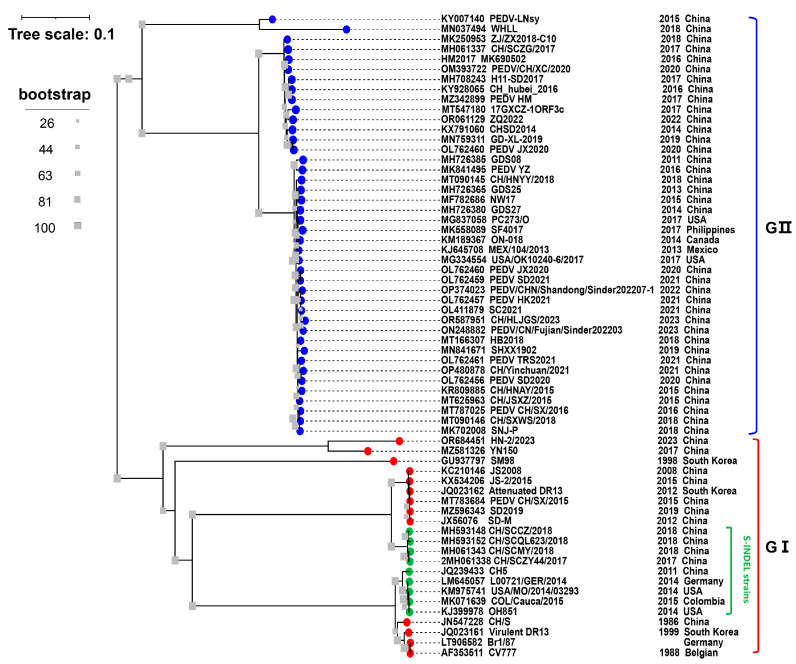
Phylogenetic analysis of the PEDVs based on S gene. The S gene sequences of 64 PEDV strains worldwide were downloaded from GenBank. The tree was constructed using MEGA 7 and visualized using the Interactive Tree Of Life (iTOL) software (version 6.8.1) (accessed on 25 October 2023). The GenBank accession numbers, strain names, collection dates, and countries are shown in the trees. The blue and red dots represent GII and GI, respectively. The green dot represents the PEDV S-INDEL strains. The gray square icon at the branch represents bootstrap values of 1000 replicates.

PEDV is classified into genotype I (GI) and GII based on the evolutionary analysis of the S gene (Figure 1). The majority of cell culture-adapted mutant strains generated by continuing passaging in vitro, such as attenuated CV777 and DR13, as well as several classical strains, including the virulent strain CV777 that was first discovered in Europe and Belgium, are classified as PEDV GI [26]. The GI strains have been reported in most of Europe and Asia, they are less virulent than other strains and typically cause sporadic outbreaks [7]. All the PEDVs isolated in China before 2010 belonged to GI, such as PEDV strain JS2008 (GenBank accession No. KC210146) (Figure 1). The main pandemic strain currently circulating in China is GII, which poses an enormous threat to the pig industry in China. In Ohio, USA, a PEDV variant OH851 strain known as PEDV S-INDEL was initially found in 2014 [12]. The S-INDEL strain was later found in China, such as the CH/SCZY44/2017 (GenBank accession No. MH593418) and CH/SCMY/2018 (GenBank accession No. MH061343) strains (Figure 1). And compared to the GI, since the PEDV S-INDEL strains differ from the PEDV GII strains in that they include two amino acid insertions (aa 161–162) and five amino acid deletions (aa 59–62, and aa 140), GII was also classified as non-S-INDEL [40]. There is a greater similarity between the S protein of S-INDEL PEDV and the GI strain.

### 2.3. PEDV Pathogenicity

PEDV can infect pigs of different ages, and the clinical symptoms are different depending on the virulence of the strain, the immune status of the herd, the age of the affected pigs, and environment variations (Table 1). The main clinical symptoms of 1- to 7-day-old newborn piglets infected with PEDV were acute watery diarrhea, dehydration, vomiting, and marked emaciation, with 80–100% mortality. Adult pigs infected with PEDV had comparatively low mortality, which was characterized by watery diarrhea, anorexia, agalactia, depression, and impaired reproductive function [41,42].

Early studies confirmed that the classic strain, PEDV CV777 (GI strain), infected piglets 1–20 days old. The clinical symptoms (diarrhea with moderate to severe) appeared at 24–40 h post-inoculation (hpi), and pathological tests also showed marked villus shortening along with the villus height [7]. Since 2010, new non-S-INDEL PEDV strains have emerged, which are highly pathogenic and are known as “highly virulent” PEDV strains based on epidemiologic and clinical data in the field. The emerging non-S-INDEL PEDV strains harbor some novel pathogenic features, such as rapid onset, rapid transmission, rapid death, and high mortality. In our investigation, 3-day-old piglets were infected with the identified novel non-S-INDEL strain PEDV CH/Yinchuan/2021, which caused vomiting and diarrhea at 18 hpi, and all the piglets died at 96 hpi (Table 1) [42]. Additionally, another study reported that the non-S-INDEL PEDV HM2017 strain caused clinical symptoms in piglets at 12 hpi, and they all died at 84 hpi [43]. In our lab, the PEDV CH/Yinchuan/2021 strain infected pig populations of all ages and caused 100% mortality in 3-day-old piglets [42]. Moreover, we found that 13-week-old growing pigs infected with PEDV experienced watery diarrhea and vomiting and significantly reduced weight gain despite low mortality (unpublished data). In the challenge test of piglets not separated from their mothers, the sows also showed anorexia after the challenge (unpublished data). Other studies have also confirmed that the highly pathogenic PEDV strains resulted in significant growth retardation in weaned pigs and significant productivity impairment, including reduced sow nursing performance, fewer total pigs and pigs born alive per litter, and lower farrowing rates [7,44]. Strikingly, the pathogenicity may change as PEDV continues to evolve; a new isolate PEDV targets not only the intestinal tract but also the respiratory system in pigs, especially the lungs [45].

**Table 1:** Clinical symptoms of PEDV-infected pigs with different genotypes.

PEDV Strain	Pig Age (Day-Old)	Onset of Clinical Signs (Hours)	Clinical Signs and Symptoms	Mortality Rate (%)	Reference
GI strain CV777	1–20	24–40	vomiting, diarrhea, and dehydration (moderate to severe)	not reported	[36]
GII strain CH/Yinchuan/2021	4	18	vomiting, watery diarrhea, lethargy, loss of appetite, huddle, and shortness of breath (severe)	100	[42]
GII strain Pintung 52	35	48–72	watery diarrhea, severe dehydration (moderate to severe)	0	[46]
GII strain CH/Yinchuan/2021	91 (fattening pig)	48	watery diarrhea, vomiting, and huddle (moderate to severe)	20	unpublished data
GII strain USA/Iowa/16465/2013	sows	72	diarrhea, vomiting, and anorexic	0	[11]
S-INDEL Iowa106	3–4	24–72	watery diarrhea and transient vomiting	18	[47]

The PEDV S-INDEL strain was initially isolated from ordinary pigs in the United States and did not cause significant clinical symptoms [12]. Relevant research on isolation, identification, and pathogenicity assessment is lacking in China, even though the PEDV S-INDEL strains have also been identified and whole-genome information is available online [48]. The S-INDEL PEDV is less virulent than highly pathogenic strains owing to several characteristics, including low piglet mortality (18%), delayed onset of clinical signs, and a shorter duration of diarrhea (Table 1) [47]. In Japan, after being infected with S-INDEL PEDV ZK-O strain, 1-week-old, specific pathogen-free (SPF) piglets showed delayed onset of PEDV fecal RNA shedding and decreased fecal viral RNA titers and fecal diarrhea scores [49]. However, outbreaks of PEDV S-INDEL in European countries caused up to 70% mortality in suckling piglets [50,51]. These data indicate that there may be regional differences in the pathogenicity of PEDV S-INDEL strains, which need to be verified by further experiments. The PEDV S-INDEL strains found in China are highly pathogenic strains in clinical features but have also not been confirmed by animal regression studies [48].

### 2.4. PEDV Transmission

PEDV mainly infects the intestinal tract of pigs, targets small intestinal epithelial cells, and then destroys intestinal villi, resulting in intestinal dysfunction in pigs, causing diarrhea, dehydration, and death, especially for newborn piglets with imperfect digestive systems [52]. Current studies have revealed that PEDV can reach the intestine and cause disease in pigs after entering PEDV-containing media through the mouth and nasal cavity [52,53]. The primary oral–fecal route of PEDV transmission involves direct or indirect contact with pigs that are clinically or subclinically infected as well as diarrheal feces and vomitus [52]. Pigs were infected with PEDV through the consumption of PEDV-contaminated food or exposure to PEDV-contaminated fomites, such as feed, feed ingredients and additives (spray-dried porcine plasma), humans (footwear and clothes), equipment, transport trailers (delivering feed, transporting pigs or carcasses), feed totes, and wild animals (mice, birds, and stray cats) [54,55,56,57,58]. A recent study reported that PEDV colonizing in the intestinal epithelial cells of sows could transfer to CD3^+^ T cells, which can transmit the virus to the mammary gland through blood circulation and ultimately deliver PEDV to the intestinal tract of piglets through colostrum to cause infection [59]. This finding indicated that sows could transmit PEDV vertically to their offspring through their milk. Additionally, utilizing semen contaminated with PEDV increases the risk of PEDV infection in the pig population [60,61]. Another important route of transmission is the fecal–nasal route, which is basically the airborne transfer of aerosolized PEDV particles into the nose to cause infection in pigs or farms [16,53]. These data indicated that PEDV infected the nasal mucosa, and then the PEDV-loaded dendritic cells transferred the virus to CD3^+^ T cells and reached the intestine through the blood circulation at the latest, resulting in infection [53].

PEDV transmission in pig populations is influenced by many factors, such as the immune status, biosafety level, and overall health status of pig populations. Feedback feeding is used to control PEDV in some pig farms in China. While this strategy can successfully lower infection, PEDV will recur as time goes on, and PEDV circulation will be formed when the overall antibody level in the pig population is reduced. In addition, although most farms are immunized with vaccines, there is a lack of real-time monitoring of antibody levels in the herd, which leads to the incorrect implementation of vaccine programming and vaccine selection. Comprehensive disinfection is necessary to prevent PED-infected farms since the virus may persist on birthing beds and other associated equipment, possibly causing secondary infections. In fattening pigs and sows, PEDV usually manifests as a subclinical disease with minimal clinical signs (diarrhea). Nevertheless, these herds keep shedding, which facilitates the transmission of PEDV to vulnerable newborn piglets and leads to a high number of fatalities. Therefore, pathogen monitoring and the removal of pathogen-infected pigs are key to the healthy breeding of the system.

Wild boars have the potential to serve as reservoirs for a variety of important infectious diseases that affect both domestic animals and people, including classical swine fever, African swine fever, leptospirosis, trichinellosis, and tuberculosis [62,63]. To date, no study has demonstrated that the wild boar acts as a reservoir for PEDV, which causes disease and even death in wild boar populations. However, a lower prevalence of PEDV in wild boars has been reported in recent research. In South Korea, 287 fecal samples were collected at random from wild boar populations by Lee et al., and 9.75% of them tested positive for PEDV. An evolutionary study revealed that the PEDV strain that was circulating in wild boars was GI, which had a high homology (97.7–100%) to the PEDV strains circulating in China [64]. Antas et al. collected 157 fecal and blood samples from wild boars in Poland to detect viral genetic material and PEDV-specific antibodies using RT-qPCR and ELISA. The results revealed that while no viral genetic material was found, 3.2% were seropositive [65]. These findings suggest that PEDV may not be fatal to wild boars or cause persistent infection, which increases the risk that the PEDV may infect domestic pigs via wild boars. There have been no reports of a comprehensive epidemiological investigation into PEDV in wild boar populations in China as of yet. Although further studies are needed to confirm whether wild boars serve as a reservoir for the PEDV [65], the risk of PEDV transmission to domestic pigs should not be undervalued.

## 3. Epidemiology of PEDV in China

The pig diarrheal disease was first recorded in 1966, it occurs seasonally, usually in the autumn and winter, and it has a localized epidemic pattern [8]. After PEDV was identified in 1984, systematic epidemiological investigations began in China [8]. A general survey of PED epidemics in some provinces, municipalities, and autonomous regions of China revealed that PED was responsible for 1.74% of the overall mortality from 36 pig diseases between 1987 and 1989, whereas TGE was responsible for 9.53% [9]. In 2004, an epidemiological investigation of PEDV in Guangxi Province showed that all pig herds had 42% morbidity and 5.69% mortality, while newborn piglets had 46.4% morbidity and 6.16% mortality, and there was 19.5% morbidity for sows, but no deaths were reported [9]. From 1984 to the beginning of 2010, PEDV was mainly sporadic or regionally endemic in China, with a low mortality rate in piglets [8]. Prior to 2010, PEDVs circulating in China belonged to GⅠ based on full-gene phylogenetic analysis [9]. Importantly, before 2010, the PEDV pandemic was effectively controlled with the widespread use of early-stage Chinese-developed inactivated tissue vaccines, inactivated CV777 vaccines, and inactivated or attenuated bivalent vaccines (PEDV and TGEV) [9].

**Figure 2:**
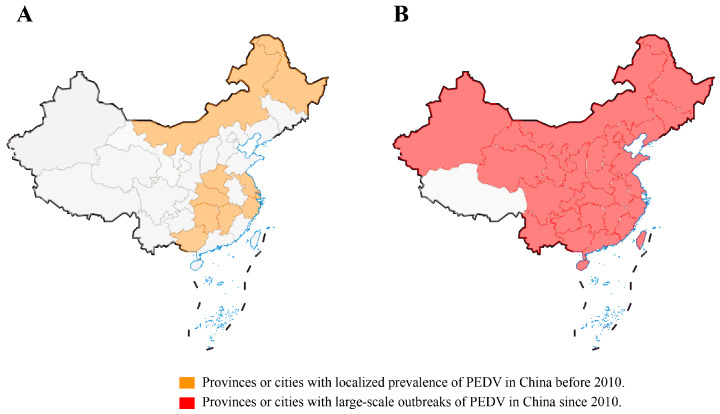
Epidemic distribution of PEDV in provinces and cities in China before and after 2010. (**A**) Provinces or cities with localized prevalence of PEDV in China before 2010. (**B**) Provinces or cities with large-scale outbreaks of PEDV in China since 2010.

In the winter of 2010, a large PED outbreak that began in southern China quickly swept throughout the nation, killing millions of piglets and catastrophically harming the pig industry (Figure 2A,B). Of note, the vaccine-immunized farms were not spared, and the mortality rate was close to 100% in neonatal piglets [10,11,38]. It has been confirmed by some investigations that PEDV has mutated in China, and the traditional vaccine PEDV CV777 cannot completely protect against highly pathogenic variants [9,10,42]. These variants have higher virulence and cause clinical symptoms in pigs of all ages, with 80–100% mortality in piglets [41,42]. In 29 Chinese provinces, excluding Tibet and Hainan, an epidemiological survey carried out between February 2011 and March 2014 found that the rate of PEDV-positive samples ranged from 61.10% to 78.49%, while the rate of PEDV-positive pig farms was 71.43% to 83.47% [9]. These data are essentially comparable with those from other countries. In 2013, data showed that piglet mortality was 90–95% and morbidity was almost 100% in the United States [11,66]. In Germany, the mortality rate for piglets infected with PEDV strains was more than 70% as of 2014 [67]. Recent studies have revealed a seropositivity of PEDV in Croatia as high as 82.8% [68]. Zhang et al. collected 149,869 clinical samples of feces and intestinal tissues from pigs from seven provinces and Shanghai City in China from 2011 to 2021 for possible pathogen identification. The results revealed that PEDV was the major causative agent, with a positive rate of more than 40%, while the positive rates of RV and PDCoV were relatively low at 1–20% and 0–14%, respectively. However, 3.21% of the samples were co-infected with PEDV, TGEV, porcine rotavirus (PoRV), PDCoV, or SADS-CoV, and 31.28% of the samples remained undiagnosed [17]. These data indicate that PEDV is the main pathogen causing PED at present, but PoRV and PDCoV cannot be ignored, and other possible pathogens need to be further identified. A recent study revealed that the Guangdong and Henan provinces are hubs for PEDV transmission in China, and the live pig trade may play a major role in disseminating the virus [69]. Since the African swine fever virus (ASFV) invaded China in 2018, the impact of PEDV on the pig industry has been underestimated, but recent statistics have revealed that PEDV is still widespread in the Chinese pig population (http://www.moa.gov.cn/was5/web/search) (accessed on 25 October 2023).

## 4. Antigenicity

Changes in the antigenic characteristics of infections to circumvent previous immunity are known as immune escape mutations. Antigenic evolution is the ongoing process by which viruses modify their antigenic characteristics [70]. As the primary target of vaccines and an immunodominant target during viral infection, the S protein has been the focus of most descriptions of the antigenic evolution of coronaviruses to date [70]. Thus, herd immunity also causes the PEDV S protein to mutate regularly, and some of these mutations alter the antigenicity of the virus to help it evade immunization [7]. The PEDVs that are the main epidemic strains are the GII strains in China, and a comparison of these strains with the vaccine strain GI (CV777) found that the S protein exhibited several insertions, deletions, and substitutions. These modifications could alter the antigenicity for GII strains, making the GI vaccine strain ineffective in preventing the PEDV large-scale epidemic caused by variants in China [9,10,48]. Nonetheless, there is relatively little data available in this regard. Lin et al. reported that prototype PEDV CV777 and three genetically different PEDV strains (virulent non-S-INDEL PEDV PC22A, S-INDEL Iowa106, and 197-DEL PC177) were investigated for two-way antigenic cross-reactivities utilizing mouse monoclonal antibody (MAb) and a panel of pig antisera against PEDV. The results showed that the convalescent-phase PEDV CV777 antisera exhibited four-fold more titers of homologous (against PEDV CV777) antibodies (cell culture immunofluorescent antibody and viral neutralizing antibody) when compared to heterologous (against U.S. PEDV strains) antibodies, and vice versa [47]. Wang and colleagues discovered that according to anti-S mouse polyclonal antibody (PAb) titers, the antigenic and serologic neutralization reactions against the S protein also revealed antigenic differences twice between the GI (PEDV CV777) and GII (highly virulent China PEDV LNCT2) [9]. In our lab, the cross-neutralization ability of GI and GII was validated through the collection of serum from sows from farms immunized with the CV777 vaccine and the preparation of mouse PAb against the S proteins of PEDV GI (CV777) and GII (CH/Yinchuan/2021). In sow serum, the anti-CV777 neutralizing antibody titers range from 32 to 512 (average titer 206). In contrast, the neutralizing CH/Yinchuan/2021 titer is 16 to 512 (average titer 145), which is approximately 1.4-fold lower than the anti-CV777 titer. Also, the neutralizing antibody titers of anti-CV777-S PAbs were significantly different from PEDV homologous (CV777) and heterologous (CH/Yinchuan/2021) strains [42]. In addition, Li et al. revealed that an MAb derived from the S1^0^ epitope of a non-S-INDEL strain could only partially or not at all cross-neutralize against PEDV S-INDEL or CV777 strains [21]. These findings suggest that mutations in the S protein of the currently circulating GII strains cause changes in its antigenicity compared to the GI vaccine strain (CV777), and the ability of the GI antisera to neutralize the GII in vitro is reduced. Although other investigations have also suggested that CV777 is unsuccessful in preventing PEDV GII strain attacks, clinical immunological challenge experiments are necessary to confirm the protective effects of the CV777 strain [9,10,42,71]. Zhang et al. reported that the inactivated PEDV CV777 vaccine can only provide limited protection against heterologous strains, and the GII-based inactivated vaccine can provide significant protection against both homologous (highly virulent CH/JX/01) and heterologous (CV777) strains [72]. In our lab, sows were immunized three times with attenuated vaccine CV777 during pregnancy. The survival rate of piglets with passive protection was only 40% after the challenge (virulent PEDV CH/Yinchuan/2021), which was lower than that of the inactivated CH/Yinchuan/2021 (80% survival rate) (unpublished data). These findings confirmed that inactivated or attenuated GI-based vaccines provide partial protection against the presently circulating variants due to alterations in the antigenicity of the GII strains. Further investigation is necessary to verify if the widespread administration of GI-based vaccines in China, either attenuated or inactivated, has contributed to the emergence of an adapted PEDV variant.

## 5. Control Strategies

### 5.1. Improve Biosecurity

Strict biosecurity measures are essential to prevent and control PEDV, especially for anything and anyone that may come into direct or indirect contact with pigs, including the provision of safe feed or feed additives and the disinfection and decontamination of transport trailers and other possibly contaminated facilities [73,74]. It was reported that the majority of contaminated feed or transport trailers were thought to be the main contaminated fomites in US swine farms during the 2013–2017 epidemic [75]. Kim et al. reported that indirect PEDV transmission via contaminated personal protective equipment infected quickly under modeled conditions. In addition, biosecurity measures such as washing exposed skin areas, taking a shower, and changing personal protective equipment can effectively reduce the risk of PEDV transmission among pig herds [74]. Fecal–oral transmission is the major route for PEDV-infected animals. Upon the invasion of a new pig farm by PEDV, perpetual circulation will ensue, potentially leading to the contamination of various elements, such as equipment, feed, and drinking water. Therefore, especially for PEDV-positive pig farms, special attention is paid to fecal treatment, such as fecal accumulation, fermentation, or disinfection, and personnel interaction and cross-use of equipment and appliances are prohibited in different pig houses. While some major pig farms already implement basic guidelines for fecal treatment and sterilization in China, many small pig farms lack biosecurity, and feces are frequently ignored as a major source of infection. In addition, pig houses should be equipped with air purification equipment as the current study confirms that aerosolized PEDV particles can be transmitted by aerosols [16,53].

### 5.2. Reasonable Immunization

The level of PEDV-specific sIgA antibodies in colostrum or milk from sows is a key factor in determining whether their offspring will be protected from PEDV infection through passive immunization [76,77]. The sIgA antibody neutralizes PEDV in the intestinal mucosa with great efficiency and is resistant to proteolytic enzymes [78]. Therefore, the key to reducing the death of susceptible piglets and controlling the PED outbreak is the reasonable immunization of pregnant sows [77]. Studies have demonstrated that the PEDV vaccines, whether inactivated or attenuated, based on the PEDV CV777 strain are poor in preventing infection against PEDV variants circulating in China [9,10]. In addition, a variant, PEDV CH/Yinchuan/2021, was isolated from farms immunized with the CV777 vaccine in our lab, and a cross-neutralization study verified that the antigenicity of CH/Yinchuan/2021 was altered due to variations in its S protein when compared to the CV777 strain, leading to a distinct neutralization profile [42]. Piglets were only partially protected against PEDV infection by prime and boost intramuscular vaccination with the inactivated PEDV vaccine due to inadequate lactogenic immunity generated in the vaccinated dams [75,79]. Our data also demonstrated that sows immunized with the inactivated variant CH/Yinchuan/2021 strain at Houhai acupoint could only partially protect piglets, exhibiting an 80% survival rate, only mild diarrhea, and reduced viral shedding after challenge, which is higher than the protection rate of CV7777 (40%) (unpublished data). Currently, as there is no attenuated vaccine licensed based on the PEDV GII strain available in China, it is essential to utilize inactivated vaccines with GII for vaccination.

Feedback feeding involves feeding the intestines or feces of PEDV-infected piglets to pregnant sows, which stimulates lactogenic immunity in sows through the gut–mammary-–sIgA axis and provides passive immunological protection to piglets against PEDV infection [75,80]. In the early stages of the PEDV invasion in the United States, whole-herd immunization of sows was carried out through feedback feeding in the absence of PEDV vaccines. This helped to control PEDV to some degree, as evidenced by a 33% increase in piglet survival, a 57% decrease in diarrhea, and a decrease in viral shedding [75]. Currently, some pig farms in China have also implemented feedback feeding as an approach to lower the prevalence of PEDV. According to the information provided by veterinarians, this method can reduce the prevalence of PEDV in pig farms, but systematic investigation data are lacking. In addition, immunizing sows with the intestinal contents of PEDV-infected piglets also had a certain protective efficiency for piglets, but no relevant data were available [9]. One study showed that the oral inoculation of virulent PEDV in sows during mid-gestation (day 57–59) induced robust immune protection, with a survival rate of 100% in piglets after challenge [76]. Although the above strategies of feedback feeding and immunizing intestinal contents or virulent PEDV strains could reduce PEDV prevalence or piglet death to some extent and are clinically easy for veterinarians to operate, there are currently no unified standards and specific operating protocols for these methods [16]. More importantly, the above measures have safety risks [81]. First, PEDV persists in pig farms. As maternal antibody levels decline, PEDV will continue to occasionally recur and may spread to other regions or neighboring pig farms. Second, there is a risk of introducing other pathogens into the farm, such as ASFV and porcine reproductive and respiratory syndrome virus (PRRSV). Hence, from a safety standpoint, it is more practical to immunize sows in China using inactivated vaccines rather than using feedback feeding; small pig farms and breeding farmers lack adequate biosafety, and feedback feeding increases the risk of PEDV spreading.

### 5.3. Accurate Monitoring

Since the causative agents of PED are currently complicated in China, it is not possible to differentiate clinically between the infection or co-infection of TGEV, PoRV, PDCoV, SADS-CoV, or PEDV. Accurate pathogen detection is essential for the deployment of preventive and control measures once PED breaks out in pig farms. For PEDV-positive pig farms, prenatal sows, delivery houses, feed, water, and equipment should be detected, and once PEDV-positive sows are found, isolation and emergency immunization should be carried out, and positive contaminants should be disinfected in time. In addition, the monitoring of antibody levels is also very critical, especially for the timely detection of antibody levels, including PEDV-specific sIgA and neutralizing antibodies in serum after vaccination. Although such work is time-consuming, it is very important for the evaluation of the vaccine immune effect and vaccination schedules.

## 6. Conclusions

Currently, large-scale PEDV epidemics are occurring in China, which poses a great threat to the pig industry due to a lack of effective vaccines. Remarkably, the inactivated or attenuated CV777 vaccine is being utilized by some farmers and pig farms in China, but studies have shown that it is poor against PEDV strains [42,71,72]. Concurrently, feedback feeding poses a risk of continuous re-infection by PEDV within herds. Although there is a licensed inactivated vaccine based on PEDV GII in China, the maternally derived antibodies cannot completely protect piglets from infection, forcing the development of a vaccine with more protection through other means. Consequently, in light of the aforementioned challenges, PEDV control has evolved into a methodical framework. The enhancement of biosafety measures, the administration of reasonable immunization, and the precise tracking of infections and antibodies are imperative to guarantee the well-being of the whole pig population.

## Data Availability

The data presented in this study are available on request from the corresponding author.

## References

[1] Drosten C., Gunther S., Preiser W., van der Werf S., Brodt H.R., Becker S., Rabenau H., Panning M., Kolesnikova L., Fouchier R.A. (2003). Identification of a novel coronavirus in patients with severe acute respiratory syndrome. N. Engl. J. Med..

[2] Zaki A.M., van Boheemen S., Bestebroer T.M., Osterhaus A.D., Fouchier R.A. (2012). Isolation of a novel coronavirus from a man with pneumonia in Saudi Arabia. N. Engl. J. Med..

[3] Sun J., He W.T., Wang L., Lai A., Ji X., Zhai X., Li G., Suchard M.A., Tian J., Zhou J. (2020). COVID-19: Epidemiology, evolution, and cross-disciplinary perspectives. Trends Mol. Med..

[4] Wang Q., Vlasova A.N., Kenney S.P., Saif L.J. (2019). Emerging and re-emerging coronaviruses in pigs. Curr. Opin. Virol..

[5] Lednicky J.A., Tagliamonte M.S., White S.K., Elbadry M.A., Alam M.M., Stephenson C.J., Bonny T.S., Loeb J.C., Telisma T., Chavannes S. (2021). Independent infections of porcine deltacoronavirus among Haitian children. Nature.

[6] Pensaert M.B., de Bouck P. (1978). A new coronavirus-like particle associated with diarrhea in swine. Arch. Virol..

[7] Lin C.M., Saif L.J., Marthaler D., Wang Q. (2016). Evolution, antigenicity and pathogenicity of global porcine epidemic diarrhea virus strains. Virus Res..

[8] Sun D., Wang X., Wei S., Chen J., Feng L. (2016). Epidemiology and vaccine of porcine epidemic diarrhea virus in China: A mini-review. J. Vet. Med. Sci..

[9] Wang D., Fang L., Xiao S. (2016). Porcine epidemic diarrhea in China. Virus Res..

[10] Li W., Li H., Liu Y., Pan Y., Deng F., Song Y., Tang X., He Q. (2012). New variants of porcine epidemic diarrhea virus, China, 2011. Emerg. Infect. Dis..

[11] Stevenson G.W., Hoang H., Schwartz K.J., Burrough E.R., Sun D., Madson D., Cooper V.L., Pillatzki A., Gauger P., Schmitt B.J. (2013). Emergence of porcine epidemic diarrhea virus in the United States: Clinical signs, lesions, and viral genomic sequences. J. Vet. Diagn. Invest..

[12] Wang L., Byrum B., Zhang Y. (2014). Detection and genetic characterization of deltacoronavirus in pigs, Ohio, USA, 2014. Emerg. Infect. Dis..

[13] Huang Y.W., Dickerman A.W., Piñeyro P., Li L., Fang L., Kiehne R., Opriessnig T., Meng X.J. (2013). Origin, evolution, and genotyping of emergent porcine epidemic diarrhea virus strains in the United States. mBio.

[14] Chen Q., Li G., Stasko J., Thomas J.T., Stensland W.R., Pillatzki A.E., Gauger P.C., Schwartz K.J., Madson D., Yoon K.J. (2014). Isolation and characterization of porcine epidemic diarrhea viruses associated with the 2013 disease outbreak among swine in the United States. J. Clin. Microbiol..

[15] Hanke D., Pohlmann A., Sauter-Louis C., Höper D., Stadler J., Ritzmann M., Steinrigl A., Schwarz B.A., Akimkin V., Fux R. (2017). Porcine epidemic diarrhea in Europe: In-detail analyses of disease dynamics and molecular epidemiology. Viruses.

[16] Jung K., Saif L.J., Wang Q. (2020). Porcine epidemic diarrhea virus (PEDV): An update on etiology, transmission, pathogenesis, and prevention and control. Virus Res..

[17] Zhang H., Zou C., Peng O., Ashraf U., Xu Q., Gong L., Fan B., Zhang Y., Xu Z., Xue C. (2023). Global dynamics of porcine enteric coronavirus PEDV epidemiology, evolution, and transmission. Mol. Biol. Evol..

[18] Kocherhans R., Bridgen A., Ackermann M., Tobler K. (2001). Completion of the porcine epidemic diarrhoea coronavirus (PEDV) genome sequence. Virus Genes.

[19] Walls A.C., Tortorici M.A., Bosch B.J., Frenz B., Rottier P.J.M., DiMaio F., Rey F.A., Veesler D. (2016). Cryo-electron microscopy structure of a coronavirus spike glycoprotein trimer. Nature.

[20] Hu Y., Xie X., Yang L., Wang A. (2021). A comprehensive view on the host factors and viral proteins associated with porcine epidemic diarrhea virus infection. Front. Microbiol..

[21] Li C., Li W., Lucio de Esesarte E., Guo H., van den Elzen P., Aarts E., van den Born E., Rottier P.J.M., Bosch B.J. (2017). Cell attachment domains of the porcine epidemic diarrhea virus spike protein are key targets of neutralizing antibodies. J. Virol..

[22] Chang C.Y., Cheng I.C., Chang Y.C., Tsai P.S., Lai S.Y., Huang Y.L., Jeng C.R., Pang V.F., Chang H.W. (2019). Identification of neutralizing monoclonal antibodies targeting novel conformational epitopes of the porcine epidemic diarrhoea virus spike protein. Sci. Rep..

[23] Chang S.H., Bae J.L., Kang T.J., Kim J., Chung G.H., Lim C.W., Laude H., Yang M.S., Jang Y.S. (2002). Identification of the epitope region capable of inducing neutralizing antibodies against the porcine epidemic diarrhea virus. Mol. Cells.

[24] Okda F.A., Lawson S., Singrey A., Nelson J., Hain K.S., Joshi L.R., Christopher-Hennings J., Nelson E.A., Diel D.G. (2017). The S2 glycoprotein subunit of porcine epidemic diarrhea virus contains immunodominant neutralizing epitopes. Virology.

[25] Cruz D.J., Kim C.J., Shin H.J. (2008). The GPRLQPY motif located at the carboxy-terminal of the spike protein induces antibodies that neutralize porcine epidemic diarrhea virus. Virus Res..

[26] Sato T., Takeyama N., Katsumata A., Tuchiya K., Kodama T., Kusanagi K. (2011). Mutations in the spike gene of porcine epidemic diarrhea virus associated with growth adaptation in vitro and attenuation of virulence in vivo. Virus Genes.

[27] Li D., Li Y., Liu Y., Chen Y., Jiao W., Feng H., Wei Q., Wang J., Zhang Y., Zhang G. (2021). Isolation and identification of a recombinant porcine epidemic diarrhea virus with a novel insertion in S1 domain. Front. Microbiol..

[28] Klumperman J., Locker J.K., Meijer A., Horzinek M.C., Geuze H.J., Rottier P.J. (1994). Coronavirus M proteins accumulate in the Golgi complex beyond the site of virion budding. J. Virol..

[29] Fan J.H., Zuo Y.Z., Shen X.Q., Gu W.Y., Di J.M. (2015). Development of an enzyme-linked immunosorbent assay for the monitoring and surveillance of antibodies to porcine epidemic diarrhea virus based on a recombinant membrane protein. J. Virol. Methods.

[30] Li C., Su M., Yin B., Guo D., Wei S., Kong F., Feng L., Wu R., Sun D. (2019). Integrin αvβ3 enhances replication of porcine epidemic diarrhea virus on Vero E6 and porcine intestinal epithelial cells. Vet. Microbiol..

[31] Shan Y., Liu Z.Q., Li G.W., Chen C., Luo H., Liu Y.J., Zhuo X.H., Shi X.F., Fang W.H., Li X.L. (2018). Nucleocapsid protein from porcine epidemic diarrhea virus isolates can antagonize interferon-λ production by blocking the nuclear factor-κB nuclear translocation. J. Zhejiang Univ. Sci. B.

[32] Wang K., Lu W., Chen J., Xie S., Shi H., Hsu H., Yu W., Xu K., Bian C., Fischer W.B. (2012). PEDV ORF3 encodes an ion channel protein and regulates virus production. FEBS Lett..

[33] Ye S., Li Z., Chen F., Li W., Guo X., Hu H., He Q. (2015). Porcine epidemic diarrhea virus ORF3 gene prolongs S-phase, facilitates formation of vesicles and promotes the proliferation of attenuated PEDV. Virus Genes.

[34] Oldham J. (1972). Letter to the editor. Pig. Farming..

[35] Chasey D., Cartwright S.F. (1978). Virus-like particles associated with porcine epidemic diarrhoea. Res. Vet. Sci..

[36] Debouck P., Pensaert M. (1980). Experimental infection of pigs with a new porcine enteric coronavirus, CV 777. Am. J. Vet. Res..

[37] Pensaert M.B., Callebaut P., Debouck P. (1982). Porcine epidemic diarrhea (PED) caused by a coronavirus: Present knowledge. Proc. Congr. Int. Pig. Vet. Soc..

[38] Wang X.M., Niu B.B., Yan H., Gao D.S., Yang X., Chen L., Chang H.T., Zhao J., Wang C.Q. (2013). Genetic properties of endemic Chinese porcine epidemic diarrhea virus strains isolated since 2010. Arch. Virol..

[39] Chen J., Liu X., Shi D., Shi H., Zhang X., Feng L. (2012). Complete genome sequence of a porcine epidemic diarrhea virus variant. J. Virol..

[40] Oka T., Saif L.J., Marthaler D., Esseili M.A., Meulia T., Lin C.M., Vlasova A.N., Jung K., Zhang Y., Wang Q. (2014). Cell culture isolation and sequence analysis of genetically diverse US porcine epidemic diarrhea virus strains including a novel strain with a large deletion in the spike gene. Vet. Microbiol..

[41] Crawford K., Lager K., Miller L., Opriessnig T., Gerber P., Hesse R. (2015). Evaluation of porcine epidemic diarrhea virus transmission and the immune response in growing pigs. Vet. Res..

[42] Lei J.L., Mia Y.Q., Guan Z., Chen H., Xiang C.H., Lu H.Q., Fang Y., Han Y., Hu R.C., Lu K.J. (2023). A porcine epidemic diarrhea virus isolated from a sow farm vaccinated with CV777 strain in Yinchuan, China: Characterization, antigenicity, and pathogenicity. Transbound. Emerg. Dis..

[43] Yang D., Su M., Li C., Zhang B., Qi S., Sun D., Yin B. (2020). Isolation and characterization of a variant subgroup GII-a porcine epidemic diarrhea virus strain in China. Microb. Pathog..

[44] Goede D., Morrison R.B. (2016). Production impact & time to stability in sow herds infected with porcine epidemic diarrhea virus (PEDV). Prev. Vet. Med..

[45] Van Diep N., Choijookhuu N., Fuke N., Myint O., Izzati U.Z., Suwanruengsri M., Hishikawa Y., Yamaguchi R. (2020). New tropisms of porcine epidemic diarrhoea virus (PEDV) in pigs naturally coinfected by variants bearing large deletions in the spike (S) protein and PEDVs possessing an intact S protein. Transbound. Emerg. Dis..

[46] Chang Y.C., Kao C.F., Chang C.Y., Jeng C.R., Tsai P.S., Pang V.F., Chiou H.Y., Peng J.Y., Cheng I.C., Chang H.W. (2017). Evaluation and comparison of the pathogenicity and host immune responses induced by a G2b Taiwan porcine epidemic diarrhea virus (strain Pintung 52) and its highly cell-culture passaged strain in conventional 5-week-old pigs. Viruses.

[47] Lin C.M., Annamalai T., Liu X., Gao X., Lu Z., El-Tholoth M., Hu H., Saif L.J., Wang Q. (2015). Experimental infection of a US spike-insertion deletion porcine epidemic diarrhea virus in conventional nursing piglets and cross-protection to the original US PEDV infection. Vet. Res..

[48] Tian Y., Yang X., Li H., Ma B., Guan R., Yang J., Chen D., Han X., Zhou L., Song Z. (2021). Molecular characterization of porcine epidemic diarrhea virus associated with outbreaks in southwest China during 2014–2018. Transbound. Emerg. Dis..

[49] Yamamoto R., Soma J., Nakanishi M., Yamaguchi R., Niinuma S. (2015). Isolation and experimental inoculation of an S INDEL strain of porcine epidemic diarrhea virus in Japan. Res. Vet. Sci..

[50] Stadler J., Zoels S., Fux R., Hanke D., Pohlmann A., Blome S., Weissenböck H., Weissenbacher-Lang C., Ritzmann M., Ladi-nig A. (2015). Emergence of porcine epidemic diarrhea virus in southern Germany. BMC Vet. Res..

[51] Mesquita J.R., Hakze-van der Honing R., Almeida A., Lourenço M., van der Poel W.H., Nascimento M.S. (2015). Outbreak of porcine epidemic diarrhea virus in Portugal, 2015. Transbound. Emerg. Dis..

[52] Jung K., Saif L.J. (2015). Porcine epidemic diarrhea virus infection: Etiology, epidemiology, pathogenesis and immunoprophylaxis. Vet. J..

[53] Li Y., Wu Q., Huang L., Yuan C., Wang J., Yang Q. (2018). An alternative pathway of enteric PEDV dissemination from nasal cavity to intestinal mucosa in swine. Nat. Commun..

[54] Pasick J., Berhane Y., Ojkic D., Maxie G., Embury-Hyatt C., Swekla K., Handel K., Fairles J., Alexandersen S. (2014). Investigation into the role of potentially contaminated feed as a source of the first-detected outbreaks of porcine epidemic diarrhea in Canada. Transbound. Emerg. Dis..

[55] Dee S., Clement T., Schelkopf A., Nerem J., Knudsen D., Christopher-Hennings J., Nelson E. (2014). An evaluation of contaminated complete feed as a vehicle for porcine epidemic diarrhea virus infection of naïve pigs following consumption via natural feeding behavior: Proof of concept. BMC Vet. Res..

[56] Opriessnig T., Xiao C.T., Gerber P.F., Zhang J., Halbur P.G. (2014). Porcine epidemic diarrhea virus RNA present in commercial spray-dried porcine plasma is not infectious to naïve pigs. PLoS ONE.

[57] Lowe J., Gauger P., Harmon K., Zhang J., Connor J., Yeske P., Loula T., Levis I., Dufresne L., Main R. (2014). Role of transportation in spread of porcine epidemic diarrhea virus infection, United States. Emerg. Infect. Dis..

[58] Jang G., Lee D., Shin S., Lim J., Won H., Eo Y., Kim C.H., Lee C. (2023). Porcine epidemic diarrhea virus: An update overview of virus epidemiology, vaccines, and control strategies in South Korea. J. Vet. Sci..

[59] Yuan C., Zhang P., Liu P., Li Y., Li J., Zhang E., Jin Y., Yang Q. (2022). A Novel Pathway for Porcine Epidemic Diarrhea Virus Transmission from Sows to Neonatal Piglets Mediated by Colostrum. J. Virol..

[60] Gallien S., Moro A., Lediguerher G., Catinot V., Paboeuf F., Bigault L., Berri M., Gauger P.C., Pozzi N., Authié E. (2018). Evidence of porcine epidemic diarrhea virus (PEDV) shedding in semen from infected specific pathogen-free boars. Vet. Res..

[61] Gallien S., Moro A., Lediguerher G., Catinot V., Paboeuf F., Bigault L., Gauger P.C., Pozzi N., Berri M., Authié E. (2019). Limited shedding of an S-InDel strain of porcine epidemic diarrhea virus (PEDV) in semen and questions regarding the infectivity of the detected virus. Vet. Microbiol..

[62] Ruiz-Fons F., Segalés J., Gortázar C. (2008). A review of viral diseases of the European wild boar: Effects of population dynamics and reservoir rôle. Vet. J..

[63] Meng X.J., Lindsay D.S., Sriranganathan N. (2009). Wild boars as sources for infectious diseases in livestock and humans. Philos. Trans. R. Soc. Lond. B Biol. Sci..

[64] Lee D.U., Kwon T., Je S.H., Yoo S.J., Seo S.W., Sunwoo S.Y., Lyoo Y.S. (2016). Wild boars harboring porcine epidemic diarrhea virus (PEDV) may play an important role as a PEDV reservoir. Vet. Microbiol..

[65] Antas M., Olech M., Szczotka-Bochniarz A. (2021). Porcine enteric coronavirus infections in wild boar in Poland—A Pilot Study. J. Vet. Res..

[66] Cima G. (2014). PED virus reinfecting U.S. herds. Virus estimated to have killed 7 million-plus pigs. J. Am. Vet. Med. Assoc..

[67] Hanke D., Jenckel M., Petrov A., Ritzmann M., Stadler J., Akimkin V., Blome S., Pohlmann A., Schirrmeier H., Beer M. (2015). Comparison of porcine epidemic diarrhea viruses from Germany and the United States, 2014. Emerg. Infect. Dis..

[68] Brnić D., Šimić I., Lojkić I., Krešić N., Jungić A., Balić D., Lolić M., Knežević D., Hengl B. (2019). The emergence of porcine epidemic diarrhoea in Croatia: Molecular characterization and serology. BMC Vet. Res..

[69] He W.T., Bollen N., Xu Y., Zhao J., Dellicour S., Yan Z., Gong W., Zhang C., Zhang L., Lu M. (2022). Phylogeography reveals association between swine trade and the spread of porcine epidemic diarrhea virus in China and across the world. Mol. Biol. Evol..

[70] Aguilar-Bretones M., Fouchier R.A., Koopmans M.P., van Nierop G.P. (2023). Impact of antigenic evolution and original antigenic sin on SARS-CoV-2 immunity. J. Clin. Investig..

[71] Gao Q., Zheng Z., Wang H., Yi S., Zhang G., Gong L. (2021). The new porcine epidemic diarrhea virus outbreak may mean that existing commercial vaccines are not enough to fully protect against the epidemic strains. Front. Vet. Sci..

[72] Zhang Y., Chen Y., Yuan W., Peng Q., Zhang F., Ye Y., Huang D., Ding Z., Lin L., He H. (2020). Evaluation of cross-protection between G1a- and G2a-genotype porcine epidemic diarrhea viruses in suckling piglets. Animals.

[73] Gordon R.K., Kotowski I.K., Coulson K.F., Link D., MacKenzie A., Bowling-Heyward J. (2019). The role of non-animal origin feed ingredients in transmission of viral pathogens of swine: A review of scientific literature. Front. Vet. Sci..

[74] Kim Y., Yang M., Goyal S.M., Cheeran M.C., Torremorell M. (2017). Evaluation of biosecurity measures to prevent indirect transmission of porcine epidemic diarrhea virus. BMC Vet. Res..

[75] Niederwerder M.C., Hesse R.A. (2018). Swine enteric coronavirus disease: A review of 4 years with porcine epidemic diarrhoea virus and porcine deltacoronavirus in the United States and Canada. Transbound. Emerg. Dis..

[76] Langel S.N., Paim F.C., Alhamo M.A., Buckley A., Van Geelen A., Lager K.M., Vlasova A.N., Saif L.J. (2019). Stage of gestation at porcine epidemic diarrhea virus infection of pregnant swine impacts maternal immunity and lactogenic immune protection of neonatal suckling piglets. Front. Immunol..

[77] Langel S.N., Wang Q., Vlasova A.N., Saif L.J. (2020). Host factors affecting generation of immunity against porcine epidemic diarrhea virus in pregnant and lactating swine and passive protection of neonates. Pathogens.

[78] Langel S.N., Paim F.C., Lager K.M., Vlasova A.N., Saif L.J. (2016). Lactogenic immunity and vaccines for porcine epidemic diarrhea virus (PEDV): Historical and current concepts. Virus Res..

[79] Gerdts V., Zakhartchouk A. (2017). Vaccines for porcine epidemic diarrhea virus and other swine coronaviruses. Vet. Microbiol..

[80] Clement T., Singrey A., Lawson S., Okda F., Nelson J., Diel D., Nelson E.A., Christopher-Hennings J. (2016). Measurement of neutralizing antibodies against porcine epidemic diarrhea virus in sow serum, colostrum, and milk samples and in piglet serum samples after feedback. J. Swine Health Prod..

[81] Stewart S.C., Dritz S.S., Woodworth J.C., Paulk C., Jones C.K. (2020). A review of strategies to impact swine feed biosecurity. Anim. Health Res. Rev..

